# A Novel Application of Risk–Risk Tradeoffs in Occupational Health: Nurses’ Occupational Asthma and Infection Risk Perceptions Related to Cleaning and Disinfection during COVID-19

**DOI:** 10.3390/ijerph192316092

**Published:** 2022-12-01

**Authors:** Amanda M. Wilson, Irene Mussio, Susan Chilton, Lynn B. Gerald, Rachael M. Jones, Frank A. Drews, Judy S. LaKind, Paloma I. Beamer

**Affiliations:** 1 Department of Community, Environment & Policy, Mel and Enid Zuckerman College of Public Health, University of Arizona, 1295 N. Martin Ave., Tucson, AZ 85724, USA; 2 Business School (Economics), Newcastle University, 5 Barrack Rd., Newcastle upon Tyne NE1 4SE, UK; 3 Population Health Sciences Program, Office of the Vice Chancellor for Health Affairs, University of Illinois at Chicago, Chicago, IL 60612, USA; 4 Department of Environmental Health Sciences, Fielding School of Public Health, University of California, Los Angeles, CA 90095, USA; 5 Department of Psychology, College of Social & Behavioral Science, University of Utah, 380 1530 E, Salt Lake City, UT 84112, USA; 6 LaKind Associates, LLC, 106 Oakdale Ave., Baltimore, MD 21228, USA; 7 Department of Epidemiology and Public Health, University of Maryland School of Medicine, 655 W. Baltimore Street, Baltimore, MD 21201, USA

**Keywords:** work-related asthma, occupational asthma, healthcare worker, risk perception

## Abstract

Background: Nurses face the risk of new onset occupational asthma (OA) due to exposures to cleaning and disinfection (C&D) agents used to prevent infections in healthcare facilities. The objective of this study was to measure nurses’ preferences when presented with simultaneous OA and respiratory viral infection (e.g., COVID-19) risks related to increased/decreased C&D activities. Methods: Nurses working in healthcare for ≥1 year and without physician-diagnosed asthma were recruited for an online anonymous survey, including four risk–risk tradeoff scenarios between OA and respiratory infection with subsequent recovery (Infect and Recovery) or subsequent death (Infect and Death). Nurses were presented with baseline risks at hypothetical “Hospital 1”, and were asked to choose Hospital 2 (increased OA risk to maintain infection risk), Hospital 3 (increased infection risk to maintain OA risk), or indicate that they were equally happy. Results: Over 70% of nurses were willing to increase infection risk to maintain baseline OA risk if they were confident they would recover from the infection. However, even when the risk of infection leading to death was much lower than OA, most nurses were not willing to accept a larger (but still small) risk of death to avoid doubling their OA risk. Age, work experience, and ever having contracted or knowing anyone who has contracted a respiratory viral infection at work influenced choices. Conclusions: We demonstrate the novel application of a risk–risk tradeoff framework to address an occupational health issue. However, more data are needed to test the generalizability of the risk preferences found in this specific risk–risk tradeoff context.

## 1. Introduction

Risk perception research indicates that individuals may be dissatisfied with the risks they face, partly because the determination of tolerable risks is made by organizations who do not represent their perspective [1]. Within health contexts, risk perceptions and acceptance thresholds are complicated by decisions that trade off risks of different outcomes. For example, cleaning and disinfection (C&D) of surfaces likely lowers infection risk, while some C&D products increase asthma-related risks in occupational contexts [2,3,4,5,6,7,8,9]. Arif and Delclos (2012) reported that 0.8% of 3650 nurse respondents had occupational asthma (OA) [3]. Other studies have shown increased odds of reported asthma among healthcare professionals who conduct general cleaning (OR = 2.02, 95% CI: 1.20, 3.40) [4] and increased relative risk of asthma for those with occupational cleaning exposure (meta-RR = 1.35, 95% CI: 1.09, 1.68) [8]. However, the true burden of OA, specifically, can be difficult to determine due to challenges in determining whether adult asthma onset is specifically due to occupational exposures. Delclos et al. (2007) reported that 6.6% of participating healthcare professionals had reported physician-diagnosed asthma with diagnosis occurring after entry into a healthcare role [4]. As C&D practices increased during the Coronavirus Infectious Disease 2019 (COVID-19) pandemic, the potential infection risk and asthma risk tradeoffs related to some C&D practices and products is unknown, despite uncertainties regarding fomite transmission risk [3,10,11,12,13]. There are also unknowns regarding how nurses view OA risks in relation to cleaning and disinfection, and how these risks are balanced with potential infection risks from unhygienic surfaces. The objective was to measure nurses’ preferences when presented with simultaneous OA and respiratory viral infection (e.g., COVID-19) risks related to increased/decreased C&D activities.

## 2. Materials and Methods

Participants were recruited from April–May 2022 via an online post and an email listserv through an Arizona-based organization for nurses. Inclusion criteria included nurses 18 years or older who (1) have worked in healthcare for at least 1 year and (2) are without physician-diagnosed asthma. Participants were compensated with a gift card to show appreciation for their time and to increase recruitment. The study procedures were approved by the University of Arizona Institutional Review Board, and participants provided informed consent. Surveys were completed anonymously. The first survey portion focused on risk–risk tradeoff questions. The second portion included demographic, risk perception, and behavioral questions.

The asthma outcome of interest was OA, defined as new onset asthma due to occupational exposure to C&D. While this study was inspired by events during COVID-19, the respiratory viral infection was merely referred to as a “respiratory viral infection” in the survey. A table was given to participants with the baseline risks for the two outcomes, described as the risks incurred from working at Hospital 1 (Table 1). Point estimates of baseline risks were first presented for the two outcomes as an expected number of cases per 100,000 people or 50 million people, depending upon the size of the risk (chosen so that ≥1 person per number of people was reported for ease of interpretation). Following a standard experimental design [14,15,16] with roots in judgement decision making [17,18], participants were then presented with the choice to opt to move from Hospital 1 to a different hospital, where they were required either to choose Hospital 2 or 3 or to indicate they were happy with either option (i.e., not able to choose Hospital 1). It should be noted that switching to a different hospital was implemented in scenarios to model previous risk–risk tradeoff surveys that utilize a change in location or “area” [15]. However, in real-world scenarios, nurses are unlikely to work at different hospitals due to C&D exposure; rather, they may change locations due to pay, leadership, workplace culture, and myriad factors.

Hospitals 2 and 3 had differing risks for the two outcomes: a risk increase in one of the two outcomes (either OA or respiratory infection) was present, while the risk for the other outcome remained the same as in Hospital 1. Following the choice of Hospital 2 or 3, participants were asked to identify how large of an increase in risk they would be willing to accept before changing their mind and selecting the other Hospital, known as the “indifference point” in economics. This is referred to herein as the respondent’s “tipping point,” implying that it serves as a threshold for the acceptable risk level.

Methods used for informing the baseline risks can be found in the Supplemental Materials. Briefly, there were four scenarios: The first two include equal baseline risks of OA and respiratory viral infection in Hospital 1, where one scenario includes infection and subsequent recovery (OA = Infect and Recover) and the other includes infection and subsequent death (OA = Infect and Death). The second two scenarios include the same outcomes, but with more realistic risks (OA > Infect and Recover and OA >> Infect and Death) (Table 2).

Descriptive statistics were used to describe participant demographics. Statistically significant (α = 0.05) differences in self-perceived willingness to take on risk, risk scoring of specific COVID-19-related and C&D-related activities, and choices in risk–risk tradeoff scenarios were evaluated across the variables, listed in Table S1, using Fisher’s exact tests. Short answer responses to rationales behind hospital choices in the risk–risk tradeoff sections were analyzed for factors influencing choice, including considerations of differences in probabilities of outcomes, preferences regarding a chronic vs. acute disease, reactions to death (OA = Infect and Death and OA >> Infect and Death scenarios), personal experiences with asthma or respiratory viral infections, and other factors mentioned by participants which not previously been considered.

Methods from behavioral economics for measuring decisions from hypothetical scenarios that pose tradeoffs between risks help to determine how individuals perceive and balance health risks, especially in cases where risks are small [14]. The objective of this study was to use a novel, interdisciplinary approach, drawing on behavioral economics to measure nurses’ perceptions and preferences regarding risk–risk tradeoffs of onset risk of occupational asthma (OA) and respiratory viral infection risks associated with fomites, where increased (decreased) C&D activities would increase (decrease) the asthma onset risk, but decrease (increase) the respiratory viral infection risk from fomites. Quantifying tolerable infection or asthma risks in a risk–risk tradeoff context is useful for prioritizing efforts to reduce a risk of one outcome that competes with another.

## 3. Results

### 3.1. Participant Demographics

Sixty-nine participants completed the survey, out of approximately 3700 members who theoretically received the email, yielding a response rate of 2%. While this is low, it should be noted that (1) recruitment of nurses for research participation during the COVID-19 pandemic has been a common challenge; (2) the main value of this study is the novel application of risk–risk tradeoff methodology in occupational health research; and 3) the demographics of our participants were similar to those of U.S. registered nurses, described in the following paragraph. The limitations of the low recruitment are further addressed in the Discussion section.

A majority of participants were female (87%), White (90%), and Non-Hispanic (93%) (Table 3). The age category with the most respondents was 41–55 years of age (41%), with 46% having 20+ years of experience in healthcare (Table 3) and 71% reporting a direct patient care role. For those conducting direct patient care, the two most common settings were hospitals (71%) and outpatient clinics (20%) (Table 3). This is comparable to the demographics of U.S. registered nurses in 2018: 87.3% female, 69% White, 61.8% working at a hospital, 35.9% ages 35–49, and 34.9% 50+ years old [19].

### 3.2. C&D Product Use and Experiences with Asthma and Respiratory Viral Infection

A full description of the results for the C&D behavioral and occupational experience survey sections can be found in the Supplemental Materials. Briefly, 96% reported using C&D products at work. Most participants (88%) reported no negative effects from C&D at work, while 12% did (cough; red eyes; skin irritation; difficulty breathing; burning sensation in the eyes, nose, and/or chest; runny nose; and headache). Sixty-seven percent reported knowing someone with asthma at or outside of work. Similar proportions of participants reported having ever (36%) or not having ever (35%) contracted a respiratory viral infection at work, or not knowing if they had (29%).

### 3.3. Risk Willingness and Perception

**Figure 1:**
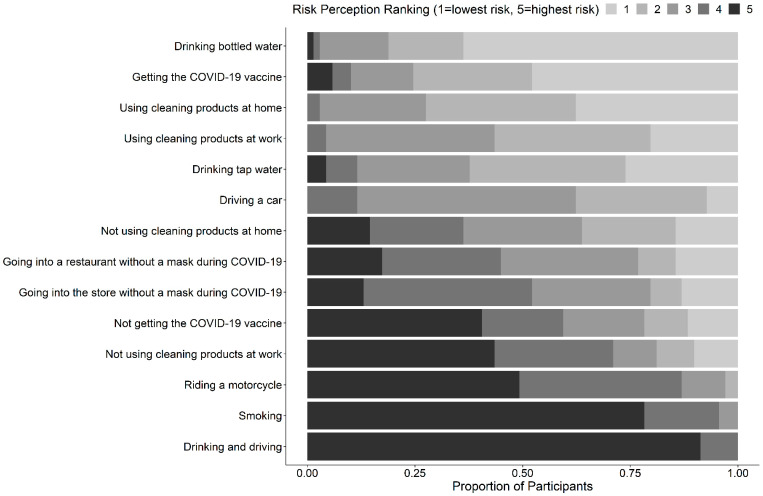
Risk perception ranking of general, COVID-19-related, and cleaning and disinfection (C&D) related activities, ordered from smallest to largest mean score from top to bottom on the *y*-axis.

On a scale of 1 (not willing to take risks) to 10 (very willing to take risks), the average score of self-reported willingness to take on risk was 5.4 (SD = 2.0). There was a significant difference in self-reported willingness to take on risks across healthcare roles (*p* = 0.003), with those in the “other” role category reporting lower risk willingness (more risk averse) than those in the direct patient care, administrative/leadership, or education roles (Figure S1). On a scale of 1 (lowest risk) to 5 (highest risk), the activity with the highest reported mean risk score was drinking and driving (mean = 4.9, SD = 0.28), followed by smoking (mean = 4.7, SD = 0.53) (Figure 1). Not receiving the COVID-19 vaccine and receiving the COVID-19 vaccine yielded mean perceived risk scores of 3.7 (SD = 1.4) and 1.9 (SD = 1.2), respectively.

### 3.4. OA = Infect and Recover Scenario

A typographical error was found in the OA = Infection and Recover scenario in the survey, where Hospital 1 specified asthma onset in 1 year, but for Hospitals 2 and 3, asthma onset was specified as 20 years, a remnant from a previously piloted version of the survey. This was present in all surveys administered, but did not influence the way in which results for this scenario were analyzed due to the scenario structure being correct, other than the specified time until asthma onset. We report the results despite the error, since these data still hold value in being the first application of risk–risk tradeoff methodology to nurse health, and offer unique insights regarding the effect of asthma onset that other scenarios do not provide. The limitation regarding this error is addressed in the Discussion.

For the OA = Infection and Recover scenario, the most common preference was Hospital 3, which maintained OA risk and increased infection risk (74%, 51/69). This choice was significantly different across years in healthcare (*p* = 0.049) and age (*p* = 0.003) (Table 4). Among participants who had been in healthcare for 1–5 years, 6–10 years, or 11–20 years, 11% (1/9), 9% (1/11), and 0% (0/17) chose to increase their OA risk to maintain their infection risk, respectively, while 31% (10/32) of those who had been in healthcare for 20+ years chose this option. Twelve percent (2/17) of those with 11–20 years of experience and 13% (4/32) of those with 20+ years of experience indicated that they were equally happy with either option (increased OA or infection), while none of the participants with 1–5 years or 6–10 years of experience chose this option. For those 18–55 years old, the largest proportion of participants chose to increase infection risk to maintain OA risk (18–30 years old: 88% (7/8), 31–40 years old: 100% (15/15), 41–55 years old: 71% (20/28)). However, for those >55 years old, a similar proportion chose to increase their OA risk to maintain their infection risk as chose to increase their infection risk to maintain their OA risk (56–65: 47% (7/15) vs. 53% (8/15), 65+: 33.3% (1/3) vs. 33.3% (1/3)).

For those who preferred increased OA risk to maintain infection risk (Hospital 2), the stated rationales included asthma manageability; expecting oneself not to be alive in 20 years, so asthma onset in that time frame is not a concern; lack of experience struggling with asthma; and concerns regarding long-term effects from COVID-19 (even though the respiratory viral infection type was not specified in the scenario). For those who preferred increased infection risk to maintain OA risk (Hospital 3), the stated rationales included dread of a chronic condition (including concerns about lifelong management), familiarity with infections at work, control over mitigation of infection risk (e.g., hand washing, mask use) and the fact that an infection would be temporary.

### 3.5. OA = Infect and Death Scenario

The most common preference was increased OA risk to maintain the risk of infection and subsequent death (83%, 57/69) (Hospital 2). Provided rationales for this choice included mitigating risk of death, the fact that asthma is manageable, lack of perceived control over infection risk mitigation, and rationalizing that death being stated as an outcome implies that the virus is “heavily circulating in the environment” and may not be a risk that one can independently mitigate. For those who chose to increase their infection and subsequent death risk to maintain asthma risk (Hospital 3), rationales included not wanting to manage a chronic illness, preferable odds (did not specify which outcome), belief about oneself being healthy and “risk of dying is low,” and possible cures for infections.

### 3.6. OA > Infect and Recover Scenario

Most participants preferred increased infection risk to maintain OA risk (Hospital 3) (91%, 63/69). There was a significant difference in preferences across age (*p* = 0.003), ever having contracted a respiratory viral infection at work (*p* = 0.003), and knowing anyone who has contracted a respiratory viral infection at work (*p* = 0.03) (Table 4). The only instances of Hospital 2 being chosen (increased asthma risk to maintain infection risk) were among participants ≥ 56 years old. Participants who had not contracted a respiratory viral infection at work more frequently selected to increase OA risk (Hospital 2) than those who had (21% 5/24 vs. 0%, 0/25). Similarly, participants who did not know anyone who had contracted a respiratory viral infection at work more frequently selected to increase OA risk (Hospital 2) than those who knew someone who had (23%, 3/13 vs. 4%, 2/55).

For those who chose to increase infection risk to maintain OA risk (Hospital 3), rationales included the fact that infection risk is still low, not wanting a chronic condition, less risk of infection than OA, and death not being an outcome of the infection. For those who chose to increase asthma risk to maintain infection risk (Hospital 2), rationales included Hospital 2 having a “similar risk profile” to the Hospital 1, the participants’ belief that they are “extremely careful” (not specifying whether this applies to potential viral exposures or C&D exposures), and fear of spreading infection to others.

### 3.7. OA >> Infect and Death Scenario

Most participants (62%, 43/69) preferred Hospital 2, where an increase in OA was taken on to maintain a baseline risk of infection and subsequent death. For those who chose to increase their OA risk to maintain their infection and subsequent death risk (Hospital 2), the rationales included choosing the option with the lowest risk of death, considerations of effects of death on family, the possibility of managing asthma, and confidence in finding solutions to reduce asthma symptoms at work. Some participants realized that the risk of death was low, but expressed why this did not drive their choice (Hospital 2: 15 out of 50 million, Hospital 3: 1500 out of 50 million risk of infection and subsequent death): “Even only 15 people, what if one of those 15 were me”.

For those who chose to increase their infection and subsequent death risk to maintain the baseline asthma risk (Hospital 3), rationales included the fact that the risk of infection and subsequent death was low; confidence in one’s immune system; effects of asthma on quality of life; the fact that death could be a risk at either hospital, regardless of asthma risk; and ability to mitigate infection risk with infection control strategies.

### 3.8. Consistency in Risk–Risk Tradeoff Choice

**Figure 2:**
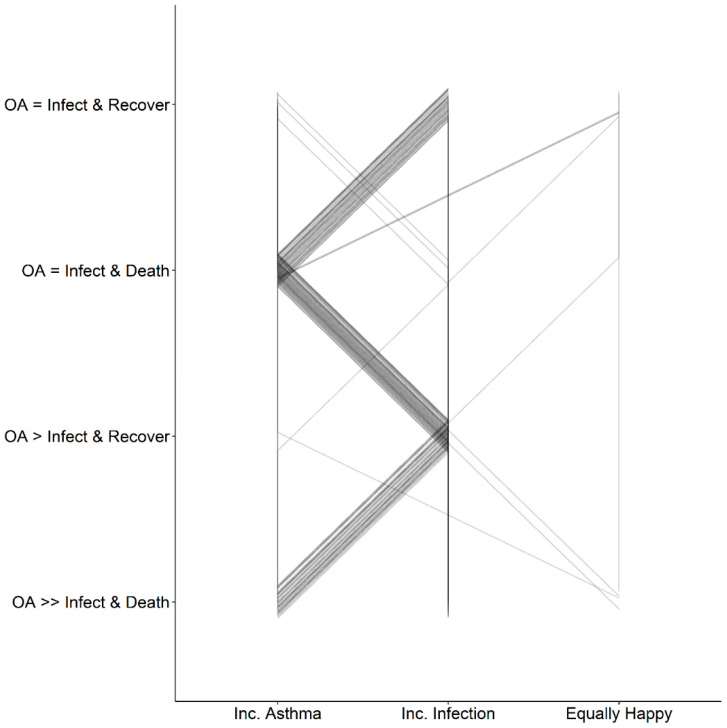
Preference choice (*x* axis) of either increased asthma risk to maintain infection risk (inc. asthma), increased infection risk to maintain asthma risk (inc. infection), or being equally happy with either increase, per individual for each scenario (in chronological order of the survey from top to bottom on the left axis). Scenarios are described based on baseline risks, either equal or unequal risks of occupational asthma (OA) and infection and recovery (infect and recover) or infection and death (infect and death).

Seven participants maintained their same preference across all scenarios: two consistently chose increased asthma, while five consistently chose increased infection (Figure 2). Forty-six percent (32/69) oscillated between increased infection and increased asthma preferences from the start to the end of the survey, choosing increased asthma whenever risk of infection was specified to end in death. Few participants were equally happy with either choice at any point in the survey (*n* = 8), and only one participant was consistently equally happy with either choice across scenarios (Figure 2).

## 4. Discussion

### 4.1. Key Findings

The majority of nurses were willing to take on an increased respiratory viral infection risk to maintain a baseline occupational asthma onset risk, if they were confident they would recover from the infection. This was true despite either equal risks or differences in respiratory viral infection and asthma onset. When the risk of respiratory viral infection and subsequent death was much lower than OA, most nurses were not willing to take on a larger, but still small, risk of death to avoid doubling their OA risk. Choices across scenarios were influenced by factors including age (OA = Infect and Recover and OA > Infect and Recover scenarios), years in healthcare (OA = Infect and Recover scenario), and ever having contracted a respiratory viral infection at work or knowing anyone who had contracted a respiratory viral infection at work (OA > Infect and Recover scenario).

Although 46% (32/69) of participants had the same choice pattern (increasing infection risk to maintain asthma risk when death was not a potential outcome of the infection, switching to increased asthma risk when death was a potential outcome of the infection) (Figure 2), there were notable differences in the amount of infection risk or asthma risk they were willing to take on (tipping point). Distributions appeared bimodal, with the largest proportions being for the smallest and largest risk choices (Supplemental Materials). Risk–risk tradeoff tipping point estimation is known for being prone to anchoring or reference point effects [15]. Anchoring describes a bias in which individuals’ decisions are biased towards the information they first received about the scenario [20]. Reference point effects exist when an individual perceives something as a gain or loss relative to a reference point, where a perceived gain may lead to risk-averse behavior and perceived loss may lead to risk-seeking behavior [21]. The use of a list to elicit the risk–risk tradeoff tipping points, as opposed to soliciting open-ended responses, may introduce such framing effects [15,22], where participants can gravitate towards middle choices or see the lower and upper bound options as “reasonable” because they are presented options [22]. While participants did not seem to gravitate towards middle responses when choosing tipping points, this may have affected the distribution of scores for self-perceived willingness to take risks, where the average score between 1 and 10 was 5.4 (SD = 2.0).

### 4.2. Limitations

Despite being the first study of its kind in occupational health, the generalizability of the data in this study is limited, considering the small sample size and small response rate from the population of interest. However, low recruitment among nurses during the COVID-19 pandemic has been a widely experienced limitation. More data should be analyzed across different types of work environments. For example, nurses working with patients who may be especially susceptible to infection (oncology or transplant patients) likely have different views regarding the importance of cleaning and disinfection than nurses with less susceptible patients. Understanding the cultural differences in hygiene and infection control across types of care will provide more context for differences in risk–risk tradeoff preferences or tipping points across care types.

It should also be noted that online surveys may present several challenges, including lack of representation for those without or with limited internet access or technology experience [23]. Additionally, those who choose to fill out the survey may encourage those they know to also participate, leading to an overrepresentation of specific views [23]. While these biases are important to consider, we also note that access to nurse populations during a pandemic via other methods may be challenging (e.g., verbal surveys in person). This is to be considered in future work in order to limit potential selection bias. Future work on risk–risk tradeoffs in a hospital context, once the pandemic is over, will likely be face-to-face as restrictions are lifted.

Another challenge due to the small sample size was low power in being able to detect differences in preference by the respiratory viral infection experience variables (having contracted a respiratory viral infection from work, knowing someone who had contracted a respiratory viral infection at work, knowing someone who had been hospitalized or died from a respiratory viral infection from work). For example, while only the OA > Infect and Recover yielded a statistically significant difference in preference type by two of these variables (ever having contracted a respiratory viral infection at work (*p* = 0.003) and knowing anyone who has contracted a respiratory viral infection at work (*p* = 0.03)), some scenarios had nearly statistically significant associations (OA = Infect and Recover, knowing someone who has died from a respiratory viral infection from work: *p* = 0.052; OA = Infect and Death, knowing someone who has contracted a respiratory viral infection from work: *p* = 0.06) (Table 4). Variability in familiarity with respiratory viral infections and the seriousness of the outcome is a potential source of bias, where experiencing or witnessing extreme outcomes from respiratory viral infections may make nurses less likely to take on infection risk, while experiences with less serious outcomes may make nurses more likely to take on infection risk. More data are needed to measure the effect which these experiences have on risk–risk tradeoff preferences, especially as COVID-19 restrictions lift and as COVID-19 becomes endemic in parts of the world.

A limitation to the interpretation of the results was the typo in the OA = Infect and Recover scenario. It is unknown whether differences between this scenario and OA > Infect and Recover are due to the latency of asthma being larger in OA = Infect and Recover than in OA > Infect and Recover, or if it was due to risk magnitude changes. Interestingly, hospital choice was significant across age groups in both scenarios. While the asthma onset was noted as having influenced the choice rationale for some participants in the OA = Infect and Recover scenario, participants who chose to increase infection risk to maintain asthma risk for either of these scenarios included dread of a chronic condition as a rationale for their choice. Future surveys should further evaluate the potential influence of asthma latency on hospital choice, as latency for long-term effects has been shown to influence risk–risk tradeoff choices [14,24], and asthma onset latency can be highly variable, on the scale of months to years [25].

A well-known challenge with risk perception surveys includes potential for preference reversals, which could explain some of the preference changes across scenarios in this study. It is possible that these changes are not truly a reflection of preference but, rather, how scenarios were presented (e.g., describing an increase in risk of infection and subsequent death as opposed to describing a decrease in their risk of not being infected and dying). One aspect to consider regarding the presentation of scenarios was the use of point values to express risk where ranges to communicate uncertainty were not used. This was conducted because the use of a numerical range may introduce bias, in that participants may gravitate towards assuming the maximum or minimum value as opposed to using the best estimate value, depending on what they believe about the likelihood of the two outcomes [26]. Visual representations may be more useful, but there is uncertainty in graphical comprehension and, therefore, regarding accurate use of the figures to influence participants’ use of the best estimate value [26].

## 5. Conclusions

To our knowledge, this is the first study that utilizes a risk–risk tradeoff survey to address respiratory health and nurses’ occupational health. While risk–risk tradeoff research has been used in transportation safety decision making [27], microbial risks from drinking water [28], and cancer risks [14,23], the application of this approach in other public health contexts is novel. The potential use of these methods to inform acceptable risks that account for competing risks resulting from an intervention will be useful in the development of local C&D policies that protect nurses at risk levels for asthma and infection which they find acceptable. While there are limitations in this study that would not warrant its use in informing policies, a larger sample size in future research will allow for a robust quantification of the magnitude of any risk premium (for avoiding one risk over the other), i.e., strength of preference over the two risks, which can be used to inform resource allocation across the prevention of the two risks. Future work is needed to investigate risk–risk tradeoffs of work-exacerbated asthma for nurses who already have asthma, and to reduce framing or anchoring effects by randomizing the scenario.

## Figures and Tables

**Table 1:** Example of baseline risk (Hospital 1) table presented to participants (OA = Infect and Recover as an example).

Hospital 1	Outcome Description	Your Risks from Fomites
Asthma onset in the next year	Lifelong condition Will require management (medication, inhalers, etc.) May be exacerbated by your job	6000 out of 100,000 people
Respiratory viral infection in the next year	60% chance of experiencing symptoms Will resolve in 2 weeks or less without long term effects	6000 out of 100,000 people

**Table 2:** Survey scenario descriptions *.

	Respiratory viral infection in the next year 60% chance of experiencing symptoms Will resolve in 2 weeks or less without long term effects	Respiratory viral infection and death in the next year Will experience symptoms Painful and difficult breathing Fatal
**First Set of Scenarios** Hospital 1 (Baseline) risks are set equal to each other at the start. Magnitudes of risk increases in outcomes for Hospitals 2 and 3 are the same.	OA = Infect and Recover **	OA = Infect and Death
**Second Set of Scenarios** Hospital 1 (Baseline) risks are not equal to each other at the start, and are more realistic. The increases in magnitudes of risk in outcomes for Hospitals 2 and 3 are not the same; rather, they depend upon the outcome.	OA > Infect and Recover	OA >> Infect and Death

* All scenarios have the same description for the asthma outcome: asthma onset in the next year, with a description: lifelong condition, will require management (medication, inhalers, etc.), may be exacerbated by your job. ** A typographical error was found in which it was stated that for Hospitals 2 and 3, the asthma onset would occur in 20 years as opposed to 1, a remnant from a previous version of the survey.

**Table 3:** Demographics of survey participants, recruited from an Arizona-based nurses association, who had worked in healthcare for at least one year and did not have physician-diagnosed asthma.

Variable		Percent (Count/69)
Gender	Male	13% (9)
	Female	87% (60)
	Nonbinary	0% (0)
	Chose not to respond	0% (0)
Race	Native Hawaiian or Other Pacific Islander	0% (0)
	Black or African American	4% (3)
	White	90% (62)
	Asian	0% (0)
	American Indian or Alaska Native	0% (0)
	More than one race	3% (2)
	Prefer not to respond	3% (2)
Ethnicity	Hispanic	4% (3)
	Not Hispanic	93% (64)
	Prefer not to respond	3% (2)
Age (years)	18–30	12% (8)
	31–40	22% (15)
	41–55	41% (28)
	56–65	22% (15)
	65+	4% (3)
Years in Healthcare	1–5	13% (9)
	6–10	16% (11)
	11–20	25% (17)
	20+	46% (32)
Role in Primary Position	Direct patient care	71% (49)
	Administrative/Leadership	7% (5)
	Education	14% (10)
	Other	7% (5)
Primary Work Setting for those in Direct Patient Care role	Hospital	51% (35)
	Outpatient clinic	14% (10)
	Home healthcare	0% (0)
	Long-term care	1% (1)
	Military	0% (0)
	School	0% (0)
	Other	4% (3)

**Table 4:** Fisher’s exact test *p*-value results of associations between preference and demographic variables, respiratory viral infection experiences, and asthma experiences. *p*-values < 0.05 are emphasized by cells that are shaded gray.

Variable	Scenario			
	OA = Infect and Recover	OA = Infect and Death	OA > Infect and Recover	OA >> Infect and Death
Age	0.003	0.38	0.003	0.82
Gender	0.46	1.00	0.58	0.25
Race	0.08	0.23	0.49	0.48
Ethnicity	0.37	0.10	1.00	0.37
Number of years working in healthcare	0.049	0.93	0.31	0.93
Healthcare role	0.45	0.40	0.23	0.97
Self-perception of willingness to take on risks	0.83	0.41	0.75	0.052
Having had negative health effects from using C&D at work	0.43	0.68	1.00	0.82
Views on what transmission route poses the greatest respiratory viral infection risk	0.73	0.68	0.83	0.62
Having ever contracted a respiratory viral infection at work	0.14	0.78	0.003	0.72
Knowing anyone who has contracted a respiratory viral infection at work	0.51	0.06	0.03	0.20
Knowing anyone who has been hospitalized due to a respiratory viral infection from work	1.00	0.66	0.36	0.78
Knowing anyone who has died due to a respiratory viral infection from work	0.052	0.13	0.62	1.00
Knowing anyone at/outside of work with asthma *	0.62	0.52	0.11	0.42

* Note that an inclusion criterion was that participants did not already have physician-diagnosed asthma, which is why this was not asked of participants.

## Data Availability

Data are available from the corresponding author upon reasonable request.

## Supplementary Materials

The following supporting information can be downloaded at: https://www.mdpi.com/article/10.3390/ijerph192316092/s1, Descriptions of baseline risk determination and infection risk calculations/estimates, detailed results of experiences with asthma and occupational infections and cleaning and disinfection behaviors, self-perceived willingness to take on risks, Tables S1–S3, Figures S1 and S2, and tipping point results. References [2,3,5,29,30,31,32,33,34,35,36,37,38,39,40,41,42] are cited in the Supplementary Materials.
