# Supplementary material for: A Novel Application of Risk–Risk Tradeoffs in Occupational Health: Nurses’ Occupational Asthma and Infection Risk Perceptions Related to Cleaning and Disinfection during COVID-19
Source: Int J Environ Res Public Health. 2022 Dec 1;19(23):16092. doi: 10.3390/ijerph192316092 (PMC9736618; doi:10.3390/ijerph192316092)
Supplement: Supplementary file 1 [file ijerph-19-16092-s001.zip › Supplemental Materials.pdf]

Supplemental Materials for

A Novel Application of Risk–Risk Tradeoffs in Occupational Health: Nurses' Occupational Asthma and Infection Risk Perceptions Related to Cleaning and Disinfection during COVID-19

Amanda M. Wilson [1,*], Irene Mussio [2], Susan Chilton [2], Lynn B. Gerald [3], Rachael M. Jones [4], Frank A. Drews [5], Judy S. LaKind [6,7] and Paloma I. Beamer [1]

[1] Department of Community, Environment & Policy, Mel and Enid Zuckerman College of Public Health, University of Arizona, 1295 N. Martin Ave., Tucson, AZ 85724, USA
[2] Business School (Economics), Newcastle University, 5 Barrack Rd., Newcastle upon Tyne NE1 4SE, UK
[3] Population Health Sciences Program, Office of the Vice Chancellor for Health Affairs, University of Illinois at Chicago, Chicago, IL 60612, USA
[4] Department of Environmental Health Sciences, Fielding School of Public Health, University of California, Los Angeles, CA 90095, USA
[5] Department of Psychology, College of Social & Behavioral Science, University of Utah, 380 1530 E, Salt Lake City, UT 84112, USA
[6] LaKind Associates, LLC, 106 Oakdale Ave., Baltimore, MD 21228, USA
[7] Department of Epidemiology and Public Health, University of Maryland School of Medicine, 655 W. Baltimore Street, Baltimore, MD 21201, USA
* Correspondence: amwilson2@arizona.edu

Contents

Description of Baseline Risk Determination 3
Description of Infection Risk Calculation 4
Description of Estimated Risk of COVID-19 and Subsequent Death 5
Cleaning and Disinfection Behaviors Results 5
Experiences with Asthma and Occupational Infections Results 6
Self-perceived Willingness to Take on Risks and General, COVID-19-related, and C&D-related Risk Perceptions Results 7
Table S1. Variables for Fisher's Exact Tests to Investigate Differences in Risk Scores and Risk-Risk Tradeoff Scenarios 8
Table S2. List of Everyday Activities in Risk Perception Questions 8
Table S3. Parameters for informing probabilities of outcomes 9
Table S4. Descriptive statistics of risk scoring for general, COVID-19 related and C&D-related activities 9
Figure S1. Distribution of scores of willingness to take risks (1=not willing, 10=very willing) by healthcare role* 10
Tipping Point Results 11

OA = Infect & Recover Scenario 11
OA = Infect & Death Scenario 11
OA > Infect & Recover Scenario 11
OA >> Infect & Death Scenario 11
Insights 11
Figure S2. Distribution of tipping points 12
References 13

Description of Baseline Risk Determination

For the first two scenarios, risks of OA and infection risk (with either recovery or death) were set equal to each other (Table 1). Risk in either outcome was changed by the same amount in the Hospital 2 and 3 choices relative to Hospital 1. The first scenario (OA = Infect & Recover) specified the outcome of the infection was recovery, while in the second scenario (OA = Infect & Death), the specified outcome for infection was death.

An assumed baseline risk of 6% for OA was informed by Delclos et al. (3) the prevalence of new onset work-related asthma among U.S. healthcare workers without asthma who engage in an average amount of cleaning and disinfection. This is similar in magnitude to other studies detailing the proportion of healthcare workers who reported having asthma onset post-hire (9). It is therefore assumed that this baseline risk of OA is for “normal” cleaning and disinfection practices. For the OA = Infect & Recover and OA = Infect & Death scenarios, this same risk was assumed for risk of infection, recovery or death. However, for OA = Infect & Death, risks were expressed as a number out of 50 million as opposed to out of 100,000, as in the OA = Infect & Recover scenario, so that the denominator was the same for OA >> Infect & Death scenario, where risk of infection and subsequent death was notably low (discussed below).

For respiratory infection risk, an annual infection risk was estimated. A risk per shift was estimated by accounting for a number of fomite touches over the course of a shift and a risk per a single contact per fomite touch (10), and aggregated over the year. This assumed an infection risk of 1×10^{-7} risk for a single fomite touch, where a hand-to-face contact directly followed a fomite touch. Cleaning and disinfection was assumed to offers a ~3 $\log_{10}$ reduction of the virus on surfaces (10), and this effect was omitted in risk calculations for scenarios with decreased cleaning and disinfection.

For all scenarios, Hospital 2 posed a twofold increased risk of OA (3), while the probability of Infect & Recover and Infect & Death did not change from Hospital 1. Hospital 3

posed an increase of risk of infection based on estimated increase in risk from fomites due to decreased cleaning and disinfection. For the OA = Infect & Death and OA > Infect & Death scenarios, the risk of death after infection was based on rates of COVID-19 mortality among U.S. healthcare workers (11). These data lacked information regarding vaccination status, meaning the risk of mortality is likely an overestimate in this study. Risk of death estimates are described below with parameter information in Table S2.

Description of Infection Risk Calculation

We assumed 30 fomite contacts would occur per care episode, informed by real-world healthcare observational data (12). However, not all of these contacts would be followed directly by a hand-to-face contact. We assumed 1% of fomite contacts would be followed directly by a hand-to-face contact. This is similar to the ratio between the frequency of hand-to-face contacts (0.4 contacts/min) (13) and the frequency of surface contacts (10 contacts/min) (12), where we assumed a smaller ratio (1% vs. 0.4/10=4%) based on anticipated less frequent hand-to-face contacts of healthcare workers in care episode settings than in classroom settings, from which the 0.4 contacts/min value originates (13). We then assumed there are 20 care episodes per shift, approximated by using data describing the number of healthcare worker entries to patient rooms per hour divided by the number of unique individuals entering, yielding entries per person per hour, times a 12 hour shift (5 entries per hour / 3 people x 12 hours = 20.4 entries per shift per person) (14). We assumed 3 shifts per week, based on a 12-hr shift schedule, and 48 working weeks per year (15). Using these values, an annual risk (P_{annual}) was calculated,

$$P_{annual} = 1 - (1 - p)^{n_f \cdot F \cdot n_c \cdot n_s \cdot n_w} \quad (1)$$

where p = a risk from a single contact with a fomite directly followed by a hand-to-face contact,

n_f= the number of fomite contacts per care episode,

F = the fraction of fomite contacts directly followed by a hand-to-face contact,

n_c= the number of care episodes per shift

n_s= the number of shifts per week

n_w= the number of working weeks per year

Other behavioral considerations such as the timing of hand hygiene relative to high-risk fomite contacts and hand-to-face contacts, the relative contamination levels of hands vs. fomites, and changes in hand-to-face and hand-to-surface contact behavior based on personal protective equipment (PPE) use are not accounted for here due to high variability and uncertainty of these parameters across anticipated scenarios.

Description of Estimated Risk of COVID-19 and Subsequent Death

The Centers for Disease Control and Prevention reported 641 deaths among 100,570 COVID-19 cases among healthcare workers. We assumed that a majority of reported cases were due to symptomatic COVID-19 infection, as the CDC states in the update on COVID-19 cases among healthcare workers that the numbers are likely underestimates due to lack of capturing asymptomatic cases, despite prioritized testing among healthcare workers (11). It was assumed that the probability of becoming symptomatic given infection $P(symptomatic|infection)$ was 60%, informed by an approximate asymptomatic proportion of 40 to 45%.(16) Therefore, $P(death|symptomatic)$ was assumed to equal 0.0064 (11).

$$P(death \cap symptomatic) = P(death|symptomatic)P(symptomatic|infection)P(infection) \quad (3)$$

Because the source of the transmission route per infection is unknown or may not be specified due to multiple transmission routes being at play, our approach inherently assumes that hospitalization and mortality risks are consistent across all transmission routes, despite some early evidence that severity of disease outcomes may be influenced by transmission route where fomite transmission routes may pose less severe disease outcome risks (17). Therefore, the estimated risk of death given infection is likely conservative for infections originating from the fomite route, specifically.

Cleaning and Disinfection Behaviors Results

All participants reported using cleaning/disinfection products at home, and 96% (66/69) reported using cleaning/disinfection products at work. The most common response for number

of times participants clean or disinfect surfaces or equipment at work was 1-3 times per day (42%, 29/69), followed by 3-5 times per day (17%, 12/69) and 10+ times per day (17%, 12/69). Wipes (n=66) were more commonly used than sprays (n=26) (fractions out of 69 are not reported since participants could have selected more than one type). A majority of participants (87%, 61/70) reported no negative effects from cleaning/disinfection at work, while 12% (8/70) did: cough; red eyes; skin irritation; difficulty breathing; burning sensation in the eyes, nose, and/or chest; runny nose; and headache. Sixty-seven percent (46/69) indicated cleaning/disinfection activities at work have changed because of COVID-19: increased frequency and number of surfaces/locations, use of disinfectants as opposed to less intense products and other product changes, increased diligence, more attention to hygiene of personal spaces at work, institute-mandated cleaning.

Experiences with Asthma and Occupational Infections Results

Sixty-seven percent (54/69) participants reported knowing someone at work or outside of work who has asthma. Similar proportions of participants reported having ever contracted a respiratory viral infection at work (36%, 25/69), not having ever contracted a respiratory viral infection at work (35%, 24/69), and not knowing if they had (29%, 29/69). For those who had, the types of infections described in 25 open-ended responses included COVID-19 (48%), cold (32%), influenza (28%), upper respiratory infections (12%), and pneumonia (4%). Most participants (45%, 31/69) believed droplets to pose the greatest risk of respiratory viral infection at work, with aerosols being the next most common response (33%, 23/69). Most participants had known someone who contracted a viral infection from work (80%, 55/69). Forty-six percent (32/69) reported having known someone who had been hospitalized for a respiratory viral infection from work, and 14% (10/70) reported having known someone who has died from a respiratory viral infection from work.

Self-perceived Willingness to Take on Risks and General, COVID-19-related, and C&D-related Risk Perceptions Results

On a scale of 1 (not willing to take risks) to 10 (very willing to take risks), the average score of self-perceived willingness to take on risk was 5.42 (SD=2.01), with a range from 1 to 10. There was a statistically significant difference in self-perceived willingness to take on risks across healthcare role (p=0.003) but was not statistically significantly different across age, ethnicity, gender, race, or years working in healthcare. Those in the “other” role category were generally less risky than those in the direct patient care, administrative/leadership, or education category. The mean score for those in administrative/leadership roles was greatest (5.8), while those in the education category had the greatest median (6.5). Distributions of scores by healthcare role can be seen in Figure S1.

On a scale of 1 (lowest risk) to 5 (highest risk), the activity with the highest mean risk score was drinking and driving (mean=4.9, SD=0.28), followed by smoking (mean=4.7, SD=0.53), and riding a motorcycle (mean=4.3, SD=0.78). Going into a restaurant or a store without a mask during COVID-19 yielded mean scores of 3.2 (SD=1.3) and 3.3 (SD=1.2), respectively. Not getting the COVID-19 vaccine and getting the COVID-19 vaccine yielded mean scores of 3.7 (SD=1.4) and 1.9 (SD=1.2), respectively. There were no statistically significant differences in the risk scoring of getting the COVID-19 or not getting the COVID-19 vaccine across age, gender, ethnicity, race, years in healthcare, or healthcare role. Both using cleaning products at work (mean=2.28, SD=0.84) and not using cleaning products at work (mean=3.86, SD=1.34) were viewed as riskier than using cleaning products at home (mean=1.9, SD=0.86) and not using cleaning products at home (mean=3.0, SD=1.27). There were no statistically significant differences in the risk scoring of using cleaning products at work or not using cleaning products at work across the variables mentioned above. Risk score distributions for activities can be seen in Figure 1.

Table S1. Variables for Fisher's Exact Tests to Investigate Differences in Risk Scores and Risk-Risk Tradeoff Scenarios

	**Variables**
Significant differences tested across these variables for self-perceived willingness to take on risk, risk scoring of specific COVID-19-related and C&D-related activities	age, gender, race, ethnicity, number of years working in healthcare, and healthcare worker role
Significant differences tested across these variables for each risk-risk tradeoff scenario decision	age, gender, race, ethnicity, number of years working in healthcare, and healthcare worker role, self-perception of willingness to take on risks, having had negative health effects from using C&D at work, views on what transmission route poses the greatest respiratory viral infection risk, having ever contracted a respiratory viral infection at work, knowing anyone who has contracted a respiratory viral infection from work, knowing anyone who has been hospitalized or died from a respiratory viral infection form work, or knowing anyone at/outside of work with asthma

Table S2. List of Everyday Activities in Risk Perception Questions

- Riding a motorcycle
- Driving a car
- Drinking and driving
- Using a gun
- Drinking tap water
- Drinking bottled water
- Smoking
- Going into a store without a mask
- Going into a restaurant without a mask
- Getting the COVID-19 vaccine
- Not getting the COVID-19 vaccine
- Using cleaning products at home
- Not using cleaning products at home

Table S3. Parameters for informing probabilities of outcomes

Parameter	Variable	Point Value	Source
Probability of infection risk from a single hand-to-fomite contact followed directly by a hand-to-face contact	p	Baseline:<br><br>Option 2 (assumes decreased cleaning/disinfection):	(10)
Number of fomite contacts per care episode	n_f	30	(12)
Fraction of fomite contacts followed by hand-to-face contacts	f_{face}	0.01	Assumed
Number of care episodes per shift	n_c	20	Assumed
Number of shifts per week	n_s	3	Assumed
Number of working weeks per year	n_w	48	Assumed
Risk of new asthma onset for healthcare workers over a year	$P(new\ asthma\ onset)$	Baseline risk: 0.06<br><br>Option 1 (Increased surface cleaning/disinfection): 0.12	(3)
Fraction of COVID-19 cases that are symptomatic	$P(symptomatic \mid infection)$	0.60	(16)
Fraction of COVID-19 cases that result in death among healthcare workers	$P(death \mid symptomatic)$	0.0064	(11)

Table S4. Descriptive statistics of risk scoring for general, COVID-19 related and C&D-related activities

**Activity**	**Mean (SD)**	**Min, Max**
Riding a motorcycle	4.3 (0.78)	2.00, 5.00
Driving a car	2.7 (0.78)	1.00, 4.00
Drinking and driving	4.9 (0.28)	4.00, 5.00
Going into a store without a mask during COVID-19	3.3 (1.2)	1.00, 5.00
Drinking bottled water	1.6 (0.91)	1.00, 5.00
Smoking	4.7 (0.53)	3.00, 5.00
Drinking tap water	2.3 (1.07)	1.00, 5.00
Going into a restaurant without a mask during COVID-19	3.2 (1.3)	1.00, 5.00
Getting the COVID-19 vaccine	1.9 (1.2)	1.00, 5.00
Not getting the COVID-19 vaccine	3.7 (1.4)	1.00, 5.00
Using cleaning products at home	1.9 (0.86)	1.00, 4.00

Not using cleaning products at home	3.0 (1.27)	1.00, 5.00
Using cleaning products at work	2.28 (0.84)	1.00, 4.00
Not using cleaning products at work	3.86 (1.34)	1.00, 5.00

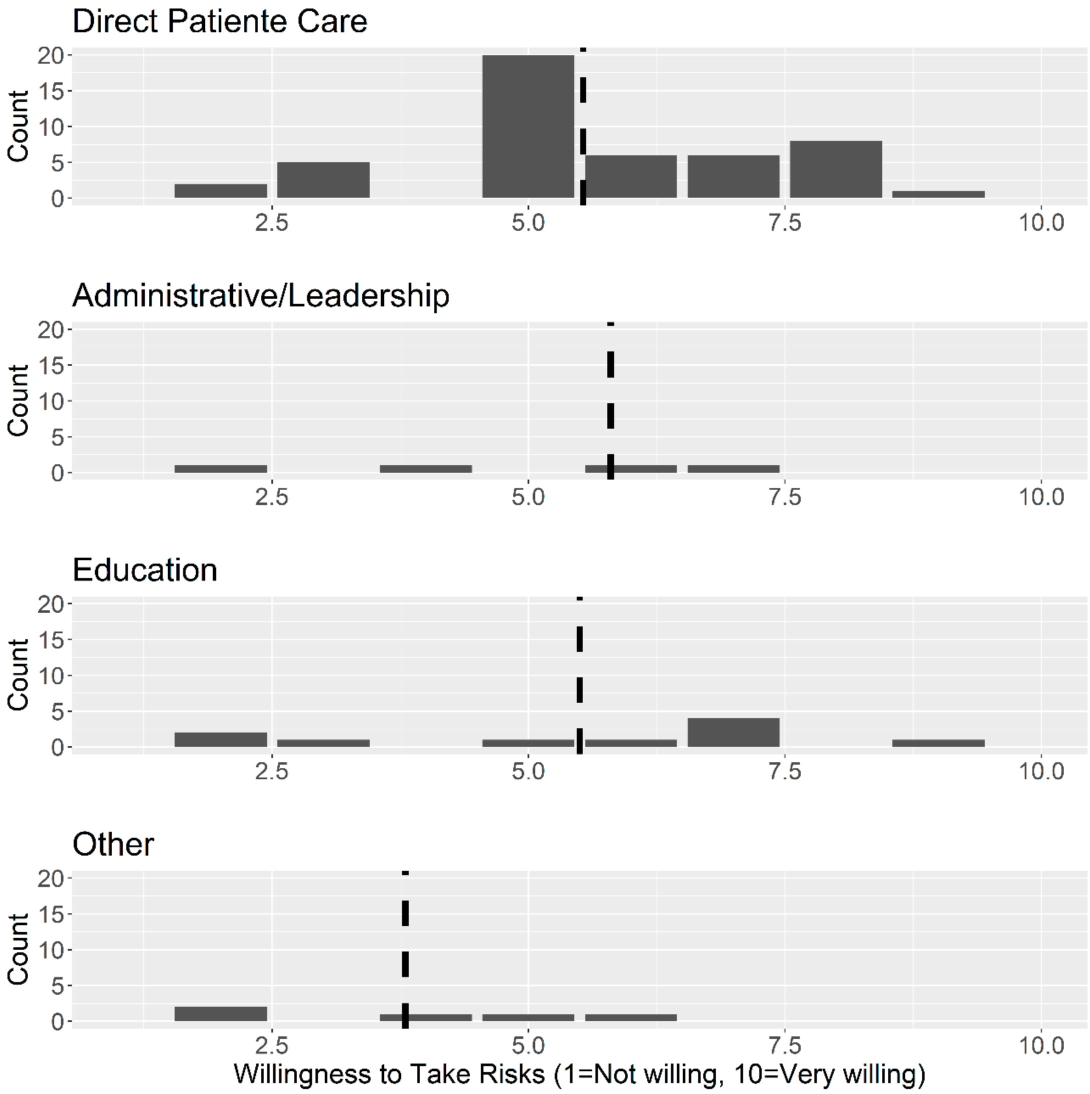

Figure S1. Distribution of scores of willingness to take risks (1=not willing, 10=very willing) by healthcare role*

*Dashed lines indicate mean willingness to take risk scores per healthcare role

Tipping Point Results

OA = Infect & Recover Scenario

For those who preferred increased OA risk to maintain infection risk (Hospital 2), only the two lowest tipping points (risk someone is willing to take on) options were selected: 12,000 or 20,000 out of 100,000. For those who preferred increased infection risk to maintain OA risk (Hospital 3), the most selected tipping points were the smallest and the largest values (12,000 or > 60,000 out of 100,000).

OA = Infect & Death Scenario

The most common preference was to increase OA risk to maintain the risk of infection and subsequent death. The most common risks of asthma that participants were willing to take to maintain their infection risk (tipping point) were the smallest and largest (6 million and > 30 million out of 50 million) options. For those who chose to increase their infection and subsequent death risk to maintain asthma risk (Hospital 3), sixty percent (6/10) selected the lowest option (6 million out of 50 million) for the infection and subsequent death risk they were willing to take on to maintain their baseline asthma risk.

OA > Infect & Recover Scenario

For those who chose to increase infection risk to maintain OA risk, the most frequently selected options for accepted infection risk to maintain baseline asthma risk were the smallest (860 out of 100,000) and largest (>60,000 out of 100,000) options. For those who chose to increase asthma risk to maintain infection risk (Hospital 2), all (n=5) selected the lowest tipping point option (12,000 out of 100,000).

OA >> Infect & Death Scenario

For those who chose to increase risk in OA to maintain a baseline risk of infection and subsequent death, the two most common risks were the smallest (6 million out of 50 million) and the largest (more than 30 million out of 50 million). For those who chose to increase their infection and subsequent death risk to maintain baseline asthma risk (Hospital 3), the most common choices were the smallest (1,500 out of 50 million) and second smallest (500,000 out of 50 million) risks.

Insights

Although 46% (32/69) of participants had the same choice pattern (increasing infection risk to maintain asthma risk when death was not a potential outcome of the infection, switching to increased asthma risk when death was a potential outcome of the infection), there were notable differences in the amount of infection risk or asthma risk they were willing to take on (tipping point), where distributions appeared bimodal with the largest proportions being for the smallest and largest risk choices.

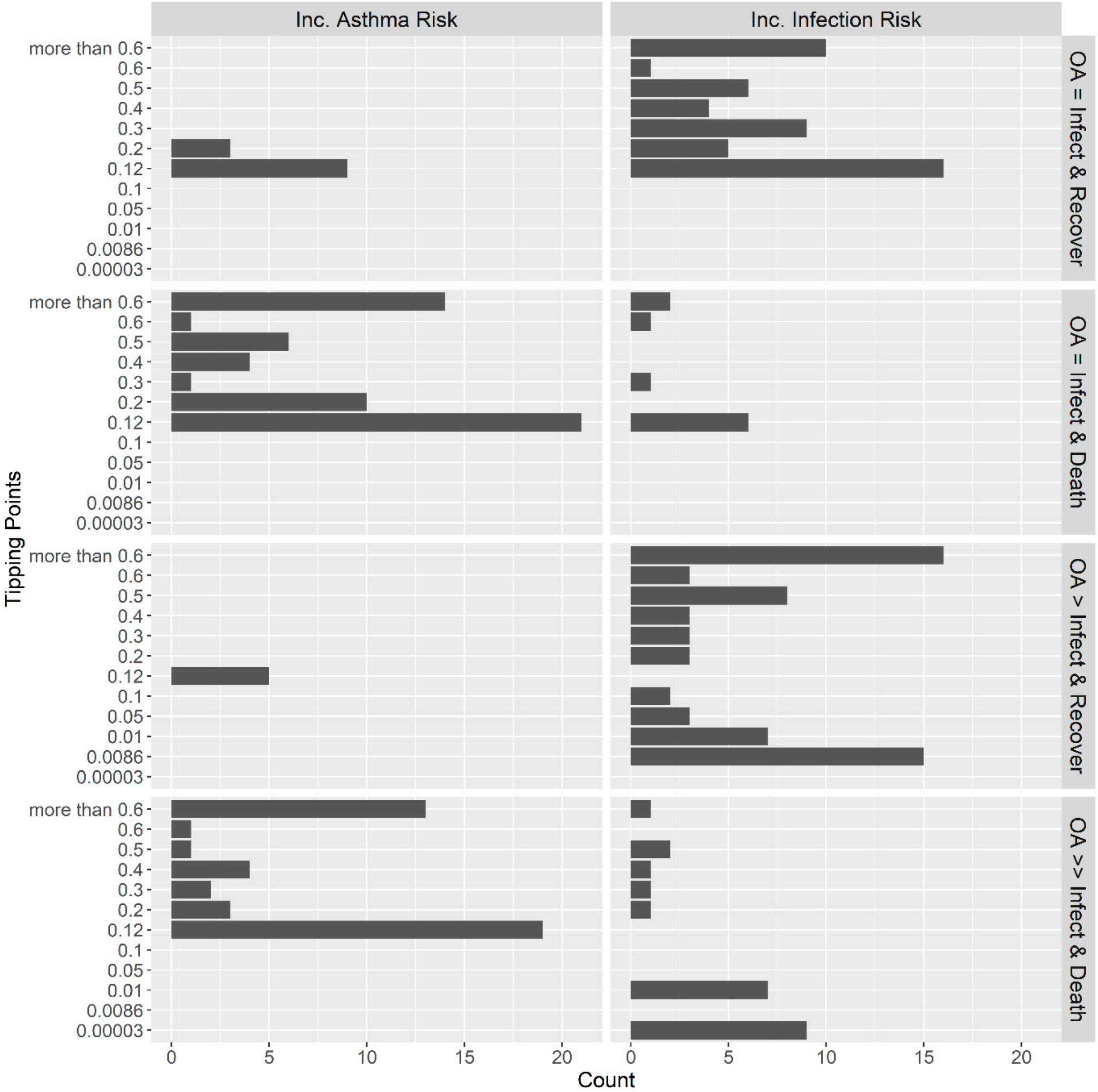

Figure S2. Distribution of tipping points (a risk someone is willing to take on for one outcome to maintain a risk for another) per scenario and per hospital choice, where scenarios are described based on baseline risks, either equal or unequal risks of occupational asthma (OA) and infection & recovery (infect & recover) or infection and death (infect & death), and hospital choices included increased asthma risk to maintain infection risk (inc. asthma risk) or increased infection risk to maintain asthma risk (inc. infection risk). As an example, a 0.2 tipping point in Inc. Asthma Risk for the OA >> Infect & Death scenario means that a participant would be willing to take on up to a 20,000 out of 100,000 risk of asthma to maintain their baseline level of infection and subsequent death.

References

1. Mwanga HH, Jeebhay MF. Work-related asthma and exposure to cleaning agents in healthcare settings – A review of the literature. Clinical Immunology. 2020;33(2):11.

2. Gonzalez M, Jégu J, Kopferschmitt MC, Donnay C, Hedelin G, Matzinger F, et al. Asthma among workers in healthcare settings: role of disinfection with quaternary ammonium compounds. Clin Exp Allergy. 2014 Mar;44(3):393–406.

3. Delclos GL, Gimeno D, Arif AA, Burau KD, Carson A, Lusk C, et al. Occupational Risk Factors and Asthma among Health Care Professionals. Am J Respir Crit Care Med. 2007 Apr;175(7):667–75.

4. Arif AA, Delclos GL. Association between cleaning-related chemicals and work-related asthma and asthma symptoms among healthcare professionals. Occup Environ Med. 2012 Jan;69(1):35–40.

5. MacKinnon M, To T, Ramsey C, Lemière C, Lougheed MD. Improving detection of work-related asthma: a review of gaps in awareness, reporting and knowledge translation. Allergy Asthma Clin Immunol. 2020 Dec;16(1):73.

6. Zheng G, Filippelli GM, Salamova A. Increased Indoor Exposure to Commonly Used Disinfectants during the COVID-19 Pandemic. Environ Sci Technol Lett. 2020 Oct 13;7(10):760–5.

7. Chang A, Schnall AH, Law R, Bronstein AC, Marraffa JM, Spiller HA, et al. Cleaning and Disinfectant Chemical Exposures and Temporal Associations with COVID-19 — National Poison Data System, United States, January 1, 2020–March 31, 2020. MMWR Morb Mortal Wkly Rep. 2020 Apr 24;69(16):496–8.

8. Centers for Disease Control and Prevention. SARS-CoV-2 and Surface (Fomite) Transmission for Indoor Community Environments [Internet]. 2021 [cited 2021 Jul 26]. Available from: https://www.cdc.gov/coronavirus/2019-ncov/more/science-and-research/surface-transmission.html

9. Caridi MN, Humann MJ, Liang X, Su FC, Stefaniak AB, LeBouf RF, et al. Occupation and task as risk factors for asthma-related outcomes among healthcare workers in New York City. International Journal of Hygiene and Environmental Health. 2019 Mar;222(2):211–20.

10. Wilson AM, Weir MH, Bloomfield SF, Scott EA, Reynolds KA. Modeling COVID-19 infection risks for a single hand-to-fomite scenario and potential risk reductions offered by surface disinfection. American Journal of Infection Control. 2021 Jun;49(6):846–8.

11. Hughes MM, Groenewold MR, Lessem SE, Xu K, Ussery EN, Wiegand RE, et al. Update: Characteristics of Health Care Personnel with COVID-19 — United States, February 12–July 16, 2020. MMWR Morb Mortal Wkly Rep. 2020 Sep 25;69(38):1364–8.

12. King MF, Wilson AM, López-García M, Proctor J, Peckham DG, Clifton IJ, et al. Why is mock care not a good proxy for predicting hand contamination during patient care? Journal of Hospital Infection. 2021 Mar;109:44–51.

13. Kwok YLA, Gralton J, McLaws ML. Face touching: A frequent habit that has implications for hand hygiene. American Journal of Infection Control. 2015 Feb;43(2):112–4.

14. Cohen B, Hyman S, Rosenberg L, Larson E. Frequency of Patient Contact with Health Care Personnel and Visitors: Implications for Infection Prevention. The Joint Commission Journal on Quality and Patient Safety. 2012 Dec;38(12):560–5.

15. Nurse Salary Guide. How Many Hours Does a Nurse Work [Internet]. 2022 [cited 2022 May 13]. Available from: https://nursesalaryguide.net/how-many-hours-does-a-nurse-work/

16. Oran DP, Topol EJ. Prevalence of Asymptomatic SARS-CoV-2 Infection: A Narrative Review. Annals of Internal Medicine. 2020 Sep 1;173(5):362–7.

17. Port, J.R.; Yinda, C.K.; Owusu, I.O.; Holbrook, M.; Fischer, R.; Bushmaker, T.; Avanzato, V.A.; Schulz, J.E.; van Doremalen, N.; Clancy, C.S.; et al. SARS-CoV-2 disease severity and transmission efficiency is increased for airborne compared to fomite exposure in Syrian hamsters. Nat Commun. 2021, 12, 4985.
