# Supplementary material for: A Novel Application of Risk–Risk Tradeoffs in Occupational Health: Nurses’ Occupational Asthma and Infection Risk Perceptions Related to Cleaning and Disinfection during COVID-19
Source: Int J Environ Res Public Health. 2022 Dec 1;19(23):16092. doi: 10.3390/ijerph192316092 (PMC9736618; doi:10.3390/ijerph192316092)
Supplement: Supplementary file 1 [file ijerph-19-16092-s001.zip › survey.pdf]

Survey Description Today we are going to ask you questions that relate to risks. A risk is the chance of something bad happening. Every day you make decisions that involve choosing between risks. For example, if you have ever crossed a road without using a pedestrian crossing to save time, you have made a decision to take a higher risk of being killed to reduce the risk of being late. Other risks include getting sick from not washing your hands in the bathroom or having a job-related accident and taking medication (which has possible side-effects).

In the following scenarios, we will ask you about choices regarding risks for two conflicting outcomes. The scenarios and outcomes are related to surface cleaning and disinfection in healthcare. Increased surface cleaning and disinfection is assumed to increase risk of asthma-related outcomes and decrease risk of respiratory viral infection.

In the following scenarios, risk of asthma onset is assumed to originate only from cleaning and disinfection in the workplace, while respiratory infection risk originates at work only from your contact with a contaminated surface followed by touching your face at work.

There is no “right” answer. We are interested in your opinions and preferences. Please answer the questions the best that you can with the information that is provided. Some scenarios will be similar but will describe a different amount of time before you will expect to experience symptoms or will describe a different level of severity of the outcome.

Benefits and Risks: This research will inform future studies aimed at understanding how healthcare workers balance the risks of these outcomes. Participants will receive a $45 Amazon gift card as compensation. This survey will take approximately 30-40 minutes to complete. It is possible that thinking about these outcomes and risks may be stressful. You may stop the survey at any time and choose to withdraw your answers.

Privacy: Your answers will be anonymized, meaning that your name will not be attached to them. At the end of the survey, you will be redirected to a link where you can enter your contact information for receiving the gift card and can optionally sign up to participate in a short interview to discuss your preferences and opinions about the survey in more detail. This contact information will not be linked to your survey.

If you have questions or concerns, please reach out to Dr. Amanda Wilson at amwilson2@arizona.edu.

Survey Pattern

There will be 6 scenarios. Each scenario will follow the same pattern of questions:

You are asked to assume that you currently work at Hospital 1, where you face the risks of two outcomes. The risks and outcome details will be described in each scenario. You will then be asked to imagine a new situation, where for whatever reason, you must move to another hospital (Hospital 2 or Hospital 3). You will be asked to make a choice between Hospital 2 and Hospital 3 under a different set of risks and outcomes. The risk and outcome details will be described in each scenario. You will then be asked to make another choice between Hospital 2 and 3 under a different set of risk and outcomes. The risk and outcome details will be described in each scenario.

Scenario 1 In hospital 1, you face the following risks.

Hospital 1	Outcome Description	Your Risk from Fomites
Asthma onset in the next year	• Lifelong condition<br>• Will require management (medication, inhalers, etc.)<br>• May be exacerbated by your job	6,000 out of 100,000 people
Respiratory viral infection in the next year	• 60% chance of experiencing symptoms<br>• Will resolve in 2 weeks or less without long term effects	6,000 out of 100,000 people

You have the option of either switching to Hospital 2 or Hospital 3, but you cannot choose to stay at Hospital 1. At Hospital 2, your risk of asthma onset increases. At Hospital 3, your risk of respiratory viral infection increases. The current situation in each Hospital is as follows:

Risks from Fomites	Hospital 2	Hospital 3
Asthma onset in 20 years	**12,000 out of 100,000**	6,000 out of 100,000
Respiratory viral infection in the next year	6,000 out of 100,000	**12,000 out of 100,000**

Which hospital option do you prefer?

◯ Hospital 2
◯ Hospital 3
◯ I am equally happy with either option.

Please explain in your own words why you chose the option that you did.

____

If you chose Hospital 2, you are willing to accept a 12,000 out of 100,000 risk for asthma onset to maintain your original risk of respiratory viral infection. What is the maximum risk you are willing to accept for asthma onset in exchange for maintaining your risk of respiratory viral infection at 6,000 out of 100,000? Choose one of the options from the dropdown menu.

◯ 12,000 out of 100,000 (no change from original answer)
◯ 20,000 out of 100,000
◯ 30,000 out of 100,000
◯ 40,000 out of 100,000
◯ 50,000 out of 100,000
◯ 60,000 out of 100,000
◯ more than 60,000 out of 100,000

If you chose Hospital 3, you are willing to accept a 12,000 out of 100,000 people risk of respiratory viral infection to maintain your original risk for asthma onset. What is the maximum risk you are willing to accept for respiratory viral infection in exchange for maintaining your risk of asthma onset at 6,000 out of 100,000? Choose one of the options from the dropdown menu.

◯ 12,000 out of 100,000 (no change from original answer)
◯ 20,000 out of 100,000
◯ 30,000 out of 100,000
◯ 40,000 out of 100,000
◯ 50,000 out of 100,000
◯ 60,000 out of 100,000
◯ more than 60,000 out of 100,000

Scenario 2 This scenario is identical to scenario 1 (same risks), except that outcome B, respiratory infection, will result in death.

In Hospital 1, you currently face the following risks.

Hospital 1	Outcome description	Your Risk from Fomites
Asthma onset in the next year	• Lifelong condition<br>• Will require management (medication, inhalers, etc.)<br>• May be exacerbated by your job	3 million out of 50 million people
Respiratory viral infection and death in the next year	• Will experience symptoms<br>• Painful and difficult breathing<br>• Fatal	3 million out of 50 million people

You have the option of either switching to Hospital 2 or Hospital 3, but you cannot choose to stay at Hospital 1. At Hospital 2, your risk of asthma onset increases. At Hospital 3, your risk of respiratory viral infection and subsequent death increases.

Risks from Fomites	Hospital 2	Hospital 3
Asthma onset in the next year	**6 million out of 50 million people**	3 million out of 50 million people
Respiratory viral infection and death in the next year	3 million out of 50 million people	**6 million out of 50 million people**

Which hospital do you prefer?

◯ Hospital 2
◯ Hospital 3
◯ I am equally happy with either option.

Please explain in your own words why you chose the option that you did.

____

If you chose Hospital 2, you are willing to accept a 6 million out of 50 million risk of asthma onset to maintain your original risk of respiratory viral infection and subsequent death. What is the maximum risk you are willing to accept for asthma onset in exchange for maintaining your risk of respiratory viral infection and subsequent death at 3 million out of 50 million? Choose one of the options from the dropdown menu.

◯ 6 million out of 50 million
◯ 10 million out of 50 million
◯ 15 million out of 50 million
◯ 20 million out of 50 million
◯ 25 million out of 50 million
◯ 30 million out of 50 million
◯ more than 30 million out of 50 million

If you chose Hospital 3, you are willing to accept a 6 million out of 50 million risk of respiratory viral infection and subsequent death to maintain your original risk of asthma onset. What is the maximum risk you are willing to accept for respiratory viral infection and subsequent death in exchange for maintaining your risk of asthma onset at 3 million out of 50 million? Choose one of the options from the dropdown menu.

◯ 6 million out of 50 million
◯ 10 million out of 50 million
◯ 15 million out of 50 million
◯ 20 million out of 50 million
◯ 25 million out of 50 million
◯ 30 million out of 50 million
◯ more than 30 million out of 50 million

Scenario 3 This scenario involves different risks for asthma onset and respiratory viral infection that does not result in death.

In Hospital 1, you face the risks of two outcomes, described below.

Hospital 1	Outcome description	Your Risk from Fomites
Asthma onset in 1 year	• Lifelong condition<br>• Will require management (medication, inhalers, etc.)<br>• May be exacerbated by your job	6,000 out of 100,000 people
Respiratory viral infection in the next year	• 60% chance of experiencing symptoms<br>• Will resolve in 2 weeks or less without long term effects	8 out of 100,000 people

You have the option of either switching to Hospital 2 or Hospital 3, but you cannot choose to stay at Hospital 1. At Hospital 2, your risk of asthma onset increases. At Hospital 3, your risk of respiratory viral infection increases.

Risks from Fomites	Hospital 2	Hospital 3
Asthma onset in 1 year	**12,000 out of 100,000 people**	6,000 out of 100,000 people
Respiratory viral infection in the next year	8 out of 100,000 people	**860 out of 100,000 people**

Which option do you prefer?

◯ Hospital 2
◯ Hospital 3
◯ I am equally happy with either option.

Please explain in your own words why you chose the option that you did.

____

If you chose Hospital 2, you are willing to accept a 12,000 out of 100,000 risk for asthma onset to maintain your original risk for respiratory viral infection. What is the maximum risk you are willing to accept for asthma onset in exchange for maintaining your risk of respiratory viral infection at 8 out of 100,000? Choose one of the options from the dropdown menu.

◯ 12,000 out of 100,000 (no change from original answer)
◯ 20,000 out of 100,000
◯ 30,000 out of 100,000
◯ 40,000 out of 100,000
◯ 50,000 out of 100,000
◯ 60,000 out of 100,000
◯ more than 60,000 out of 100,000

If you chose Hospital 3, you are willing to accept a 860 out of 100,000 risk for respiratory viral infection to maintain your original risk for asthma onset. What is the maximum risk you are willing to accept for respiratory viral infection in exchange for maintaining your risk of asthma onset at 6,000 out of 100,000? Choose one of the options in the dropdown menu.

◯ 860 out of 100,000 (no change from original answer)
◯ 1,000 out of 100,000
◯ 5,000 out of 100,000
◯ 10,000 out of 100,000
◯ 20,000 out of 100,000
◯ 30,000 out of 100,000
◯ 40,000 out of 100,000
◯ 50,000 out of 100,000
◯ 60,000 out of 100,000
◯ more than 60,000 out of 100,000

Scenario 4 This scenario involves different risks for asthma onset and respiratory viral infection that results in death.

In Hospital 1, you face the risks of two outcomes, described below.

Hospital 1	Outcome description	Your Risk from Fomites
Asthma onset in the next year	• Lifelong condition<br>• Will require management (medication, inhalers, etc.)<br>• May be exacerbated by your job	3 million out of 50 million people
Respiratory viral infection and death in the next year	• Will experience symptoms<br>• Painful and difficult breathing<br>• Fatal	15 out of 50 million people

You have the option of either switching to Hospital 2 or Hospital 3, but you cannot choose to stay at Hospital 1. At Hospital 2, your risk of asthma onset increases. At Hospital 3, your risk of respiratory viral infection and subsequent death increases.

Risks from Fomites	Hospital 2	Hospital 3
Asthma onset in the next year	**6 million out of 50 million people**	3 million out of 50 million people
Respiratory viral infection and death in the next year	15 out of 50 million people	**1,500 out of 50 million people**

Which option do you prefer?

◯ Hospital 2
◯ Hospital 3
◯ I am equally happy with either option.

Please explain in your own words why you chose the option that you did.

____

If you chose Hospital 2, you are willing to accept a 6 million out of 50 million risk of asthma onset to maintain your original risk for respiratory viral infection and subsequent death. What is the maximum risk you are willing to accept for asthma onset in exchange for maintaining your risk of respiratory viral infection and subsequent death at 15 out of 50 million? Choose one of the options from the dropdown menu.

◯ 6 million out of 50 million
◯ 10 million out of 50 million
◯ 15 million out of 50 million
◯ 20 million out of 50 million
◯ 25 million out of 50 million
◯ 30 million out of 50 million
◯ more than 30 million out of 50 million

If you chose Hospital 3, you are willing to accept a 1,500 out of 50 million risk for respiratory viral infection and subsequent death to maintain your original risk of asthma onset. What is the maximum risk you are willing to accept for respiratory viral infection and subsequent death in exchange for maintaining your risk of asthma onset at 3 million out of 50 million? Choose one of the options from the dropdown menu.

◯ 1,500 out of 50 million (no change from original answer)
◯ 500,000 out of 50 million
◯ 2.5 million out of 50 million
◯ 5 million out of 50 million
◯ 10 million out of 50 million
◯ 15 million out of 50 million
◯ 20 million out of 50 million
◯ 25 million out of 50 million
◯ 30 million out of 50 million
◯ more than 30 million out of 50 million

This section pertains to how you perceive risk. Risk is an evaluation of both a hazard and the likelihood that it will occur ("could this hurt me in some way, and how likely is it to hurt me in some way"). We are specifically interested in how you perceive the risk of certain activities today - which might be the same or different from last week, month, or year.

I am going to give you a list of activities and will ask you how risky you feel they are. I am not asking what you think other people feel about the activities, but rather what you think about them in your own life. Please note that you do not have to have participated in any of the activities in order to give your opinion of their level of risk.

Please rate the following activities on a 1-5 scale in terms of how risky you feel it is with 1 being something you consider very low risk and 5 being something you consider extremely risky.

	1	2	3	4	5
Riding a motorcycle	○	○	○	○	○
Driving a car	○	○	○	○	○
Using cleaning products at work	○	○	○	○	○
Drinking and driving	○	○	○	○	○
Not getting the COVID-19 vaccine	○	○	○	○	○
Using a gun	○	○	○	○	○
Using cleaning products at home	○	○	○	○	○
Going into the store without a mask during COVID-19	○	○	○	○	○
Drinking bottled water	○	○	○	○	○
Smoking	○	○	○	○	○
Drinking tap water	○	○	○	○	○
Going into a restaurant without a mask during COVID-19	○	○	○	○	○
Not using cleaning products at work	○	○	○	○	○
Getting the COVID-19 vaccine	○	○	○	○	○
Not using cleaning products at home	○	○	○	○	○

How do you see yourself: are you generally a person who is fully prepared to take risks, or do you try to avoid taking risks? Please choose a value on a scale from 1 to 10, where 1 means 'not willing to take risks' and 10 means 'very willing to take risks."

◯ 1 ◯ 2 ◯ 3 ◯ 4 ◯ 5 ◯ 6 ◯ 7 ◯ 8 ◯ 9 ◯ 10

Cleaning and Disinfection Experiences

Do you use cleaning and/or disinfection products at home?

◯ Yes ◯ No ◯ Choose not to respond

Do you use cleaning and/or disinfection products at work?

◯ Yes ◯ No ◯ Choose not to respond

Have your cleaning and/or disinfection activities at work changed because of COVID-19?

◯ Yes ◯ No ◯ Choose not to respond

If yes, please explain.

Work-related Cleaning Questions

How often do you clean or disinfect surfaces or equipment at work?

◯ < 1 times a day ◯ 1-3 times a day ◯ 3-5 times a day ◯ 5-10 times a day ◯ 10+ times a day
◯ Choose not to respond

How many shifts a week do you work?

____

What is the average number of hours per shift?

____

What type of product do you use to disinfect/clean surfaces? (select all that apply)

☐ Spray ☐ Wipe ☐ None ☐ Other ☐ Choose not to respond

Do you experience any negative health effects from cleaning and disinfection activities in the workplace?

◯ Yes ◯ No ◯ Choose not to respond

If yes, what negative health effects do you experience?

Do you know anyone who has asthma at work or outside of work?

◯ Yes ◯ No ◯ Choose not to respond

Occupational Infections

In your view, what poses the greatest risks of respiratory viral infection at work?

◯ Fomites (germs on surfaces) ◯ Aerosols ◯ Droplets ◯ Other ◯ Choose not to respond

If you selected "other," please list your answer here.

Have you ever contracted a respiratory viral infection from work?

◯ Yes ◯ No ◯ I don't know ◯ Choose not to respond

If yes, what was it?

	Yes	No	Choose not to respond
Do you know anyone who has contracted a respiratory viral infection from work?	◯	◯	◯
Do you know anyone who has been hospitalized for a respiratory viral infection due to an infection from work?	◯	◯	◯
Do you know anyone who has died from a respiratory viral infection due to an infection from work?	◯	◯	◯

Demographic Questions

What gender do you identify with?

○ Male ○ Female ○ Non-binary ○ Choose not to respond

What is your ethnicity?

○ Hispanic ○ Not Hispanic ○ Don't know ○ Prefer not to answer

What is your race?

○ American Indian/Alaska Native ○ Asian ○ Native Hawaiian or Other Pacific Islander
○ Black or African American ○ White ○ More than One Race ○ Don't know ○ Prefer not to answer

What is your age?

○ 18-25 ○ 26-30 ○ 31-35 ○ 36-40 ○ 41-45 ○ 46-51 ○ 51-55 ○ 56-65 ○ 65+
○ Choose not to respond

How many years have you worked in healthcare?

○ < 1 ○ 1-5 ○ 6-10 ○ 11-20 ○ 20+ ○ Choose not to respond

What is your role in your primary position?

○ Direct patient care
○ Administrative/leadership
○ Education
○ Other

What is your primary work setting?

○ Hospital
○ Outpatient clinic
○ Home Healthcare
○ Long-term Care
○ Military
○ School
○ Other

Final Question

Is there anything else you would like to share about your experiences or concerns regarding microbial exposures, asthma risks, or cleaning and disinfection protocols in the workplace?
